# Supplementary material for: Dimensionality of locomotor behaviors in developing C. elegans
Source: PLoS Comput Biol. 2024 Mar 4;20(3):e1011906. doi: 10.1371/journal.pcbi.1011906 (PMC10939432; doi:10.1371/journal.pcbi.1011906)
Supplement: S1 Table — (PDF) [file pcbi.1011906.s006.pdf]

Table of Definitions	
Kymograph	A graphical representation of postures (y-axis) over time (x-axis), where the angle between segments is represented in a blue-white-red color scheme. Rhythmic locomotion is seen as repeating striped patterns, whereas unstructured flailing lacks stripes.
Eigenworm	Mathematically, these are the eigenvectors of the postural covariance matrix. Their shape represents common postures of the worm during locomotion.
Eigenworm amplitudes	The scalar associated with each eigenworm when reconstructing the original posture.
Eigenvalue	Describes the amount of variance of the original data that is captured by an eigenvector
Dimensionality	The effective number of degrees-of-freedom present in a data set. The lower bound is 1, the upper bound is the number of recorded features (N), with most data containing correlations that reduce the dimensionality below N. The participation ratio (PR) is a continuous estimate of dimensionality derived from the eigenvalues that estimates how many dimensions are required to describe approximately 80% to 90% of the variance. [54]
Cumulative Variance Explained	A cumulative sum of the eigenvalues, typically expressed as a ratio or percentage, describing how much variance in the original data is captured by the eigenvectors.
Kernel Density Estimate	An estimate of the probability distribution based on kernel smoothing the histogram of a finite sample of data.

Naturalistic	“behaviors that are representative of actions generated during real-world tasks, like exploring new environments, obtaining food, finding shelter, and identifying mates; naturalistic behaviors … are also largely self-motivated and expressed freely without physical restraint.” [55]
