# Supplementary material for: Dimensionality of locomotor behaviors in developing C. elegans
Source: PLoS Comput Biol. 2024 Mar 4;20(3):e1011906. doi: 10.1371/journal.pcbi.1011906 (PMC10939432; doi:10.1371/journal.pcbi.1011906)
Supplement: Attachment — Submitted filename: Response to Reviewers.pdf [file pcbi.1011906.s007.pdf]

Responses to the review comments (Hassinan, Sterrett et al., PCOMPBIOL-D-23-01560)

We appreciate the time and effort of the reviewers and editors in considering our manuscript. We have addressed all issues indicated in the review report, and our responses are outlined below.

Reviewer #2:

1. The table of definitions is unnecessary as these terms are mostly described already in the text. Perhaps put it in the supplement if anything.

As suggested by the reviewer, we have moved the table of definitions to the supplemental information and made minor text changes to citations - switching "Table 1" to "S1 Table". (Lines 130, 220 and 723 in the revised manuscript).

2. Is there a reason 11 segments is the right number or was it just used for convenience to have 10 angles to look at? Has there been any work done to optimize the number of segments to maximize the variation captured by the fewest eigenworms or anything along those lines?

We analyzed a small, held-out set of the postural data using 5, 9, 11, 17, and 33 segments. Our findings show that the eigenworm analyses with 11, 17, and 33 segments yielded identical results, indicating that 11 segments are sufficient to capture the necessary postural variation. Consequently, we chose to use 11 segments for the remainder of our analyses in the paper. We have now included revised text to clarify this point in the methods section. (Lines 385-387 in the revised manuscript).

3. Line 177 - Does the constant density in eigenworm space show that it's a constant propagating wave along the worm? I think it shows that the worm goes constantly between eigenworm one and eigenworm two states which seem to have slightly different wavelengths, ~1.5 waves per worm and ~2 waves per worm. From the videos of course it looks like a constant propagating wave I'm just not sure that is what this plot directly shows and there may be a step missing in the logic here that could be elaborated on.

We agree that constant density alone does not adequately support the claim of a constant propagating wave along the worm. Therefore, we conducted an analysis of the phase speed and observed it remains constant across the phase in the ring, as illustrated in Fig 2f. We hope clarification addresses the confusion.

4. Line 183 / Figure 2g - You say that the first four eigenworms in the transition assay match the first two for each crawling and swimming locomotion. It strikes me that plotting the crawling and swimming rings in the space of the first three eigenworms is missing out on one of the eigenworms for one type of locomotion. Instead perhaps you can make two plots with these two rings on them one for the crawling eigenworms and one for the swimming ones? For example if eigenworms one and three are the same as the crawling eigenworms and two and four are those of swimming can you make two 2D plots with both swimming and crawling locomotion rings on them. One plot that is eigenworm one vs. eigenworm three and one that is eigenworm two vs. eigenworm four. Then I would expect that the orange crawling points would trace out a nice circle in the eigenworm one vs. eigenworm three plot while they blue points would be more scattered (?) in this space, and vice versa.

Following the reviewer's suggestion, we have incorporated a supporting figure (S1 Figure) that presents the transition assay in two separate 2D plots – one plotting eigenworm 1 against eigenworm 2 (the swimming eigenworms plane), and the other plotting eigenworm 3 against eigenworm 4 (the crawling eigenworms plane). These plots show that the two locomotion gaits trace separate rings in this four-dimensional space. We propose retaining the three-dimensional presentation in the main figure to succinctly demonstrate this observation, while providing the two 2D plots in Supporting Information for enhanced clarity. (Lines 184 and 711-714 in the revised manuscript).

5. Line 218 - I am slightly confused by the description of PR. Figure 1d seems to show that 85% of the variation is captured by the first two eigenworms, wouldn't that make the PR for adult worms 2 or less since it is already above 80% there?

We thank the reviewer for pointing out this discrepancy. We have updated the definition of participation ratio to include the range of possible values (80% to 90% of variance explained), aligning it with our data. (Lines 219 and 413, and in the supporting table 1 on line 723).

6. ~Line 235 - Do the worms grow uniformly along their length? Since the ancestry of all the cells in adult worms is known can you segment the different growth stages at the same cell rather than at equally spaced points?

Previous research [1] has shown that the *C. elegans* brain grows uniformly across its development. Technically, segmenting worms at specific cells would require visible and cell-specific markers, necessitating complex setups and extensive labor.

Our results also suggest that developmental changes in body length are not the determining factor for eigenworm shapes. From the late L1 to day-1 adult stages, the worms grow from approximately 0.25 mm to 1 mm. Despite a roughly 4-fold increase in body length, crawling eigenworm postures are maintained into adulthood. In contrast, between the young and late L1 stage, worms grow from 0.2 to 0.25 mm, a 1.25-fold increase, yet we see deviations in crawling eigenworms. Collectively, these data indicate that changes in eigenworm shapes are not due to non-uniform growth in body length.

References:

1. Witvliet D, Mulcahy B, Mitchell JK, Meirovitch Y, Berger DR, Wu Y, et al. Connectomes across development reveal principles of brain maturation. Nature. 2021 Aug 12 Sep 28;596(7871):257–61.
